# BOOK NOTICES

**DOI:** 10.3402/ijch.v72i0.21032

**Published:** 2013-05-14

Robert Fortuine. *Alaska Native Medical Center: A History 1953–1983*. Anchorage: Alaska Native Medical Center, 1986. 2^nd^ printing 2012

**Figure:**
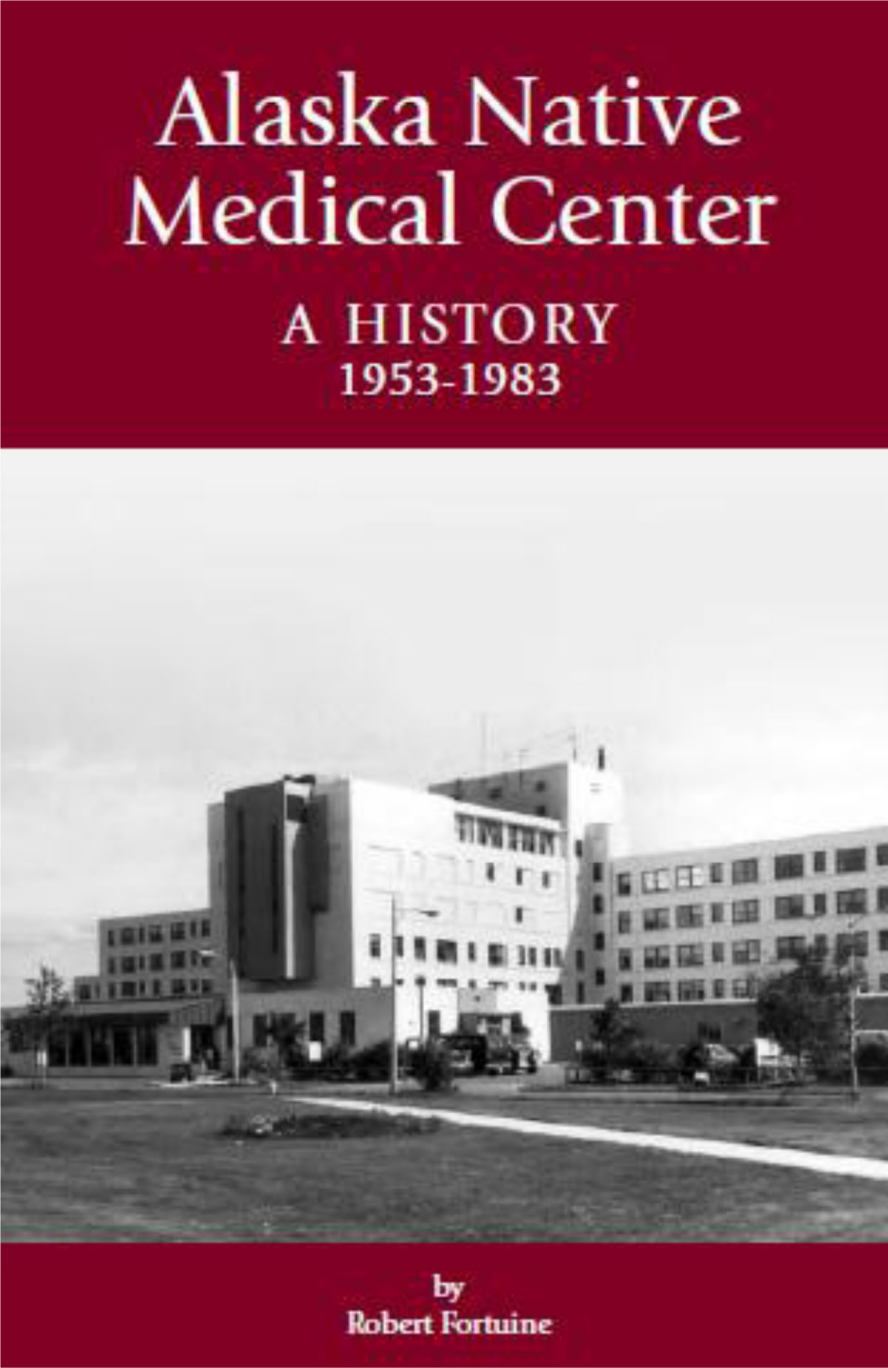


The late Dr. Robert Fortuine (1934–2009) had a distinguished career in Alaska as a Commissioned Officer of the US Public Health Service. His passion was medical history, and he published several books on the history of health and health care in Alaska.

Long out-of-print and unavailable, this history of the ANMC was written to commemorate the 30^th^ anniversary of the hospital. Now, through the efforts of Susan Clift and Patricia Hackley, and financial support from the Alaska Native Tribal Health Consortium, the ANMC Auxiliary, and many individuals, a second printing has been published online. It can be purchased from Lulu.com:

http://www.lulu.com/shop/search.ep?type=Print+Products&keyWords=fortuine&sitesearch=lulu.com&q=&x=10&y=8

T. Kue Young, Rajiv Rawat, Winfried Dallmann, Susan Chatwood, and Peter Bjerregaard, editors. *Circumpolar Health Atlas*. Toronto: University of Toronto Press [ISBN 978-1-4426-4456-4]

**Figure:**
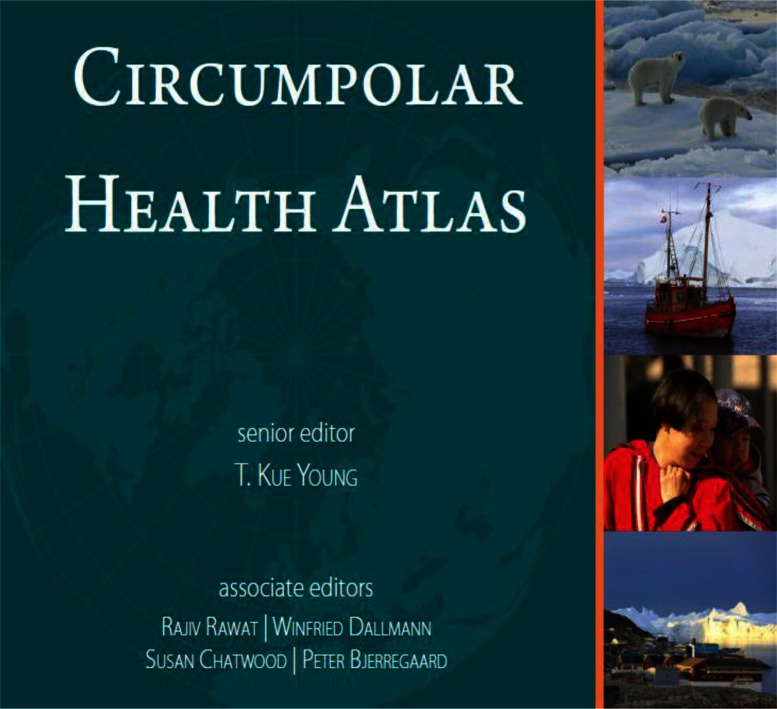


This is an atlas about the health of the diverse populations who inhabit the circumpolar regions in the northern hemisphere. As an atlas, it uses maps, charts, tables, and images to describe and explain visually the major health patterns and related issues. The editors define and conceptualize “health” very broadly, and it is also their conviction that health researchers, service providers, and policy makers working in the North and for the North need a broad and multidisciplinary understanding of northern conditions to put health into its proper context. There are 5 parts: (1) The Circumpolar World; (2) Circumpolar Peoples; (3) Health Status; (4) Health Determinants; and (5) Health Systems.

The book can be purchased from the University of Toronto Press and major online booksellers:

http://www.utppublishing.com/Circumpolar-Health-Atlas.html

Laurie Meijer Drees. *Healing Histories: Stories from Canada's Indian Hospitals*. Edmonton: University of Alberta Press, 2013 [ISBN 978-0-88864-650-7]

**Figure:**
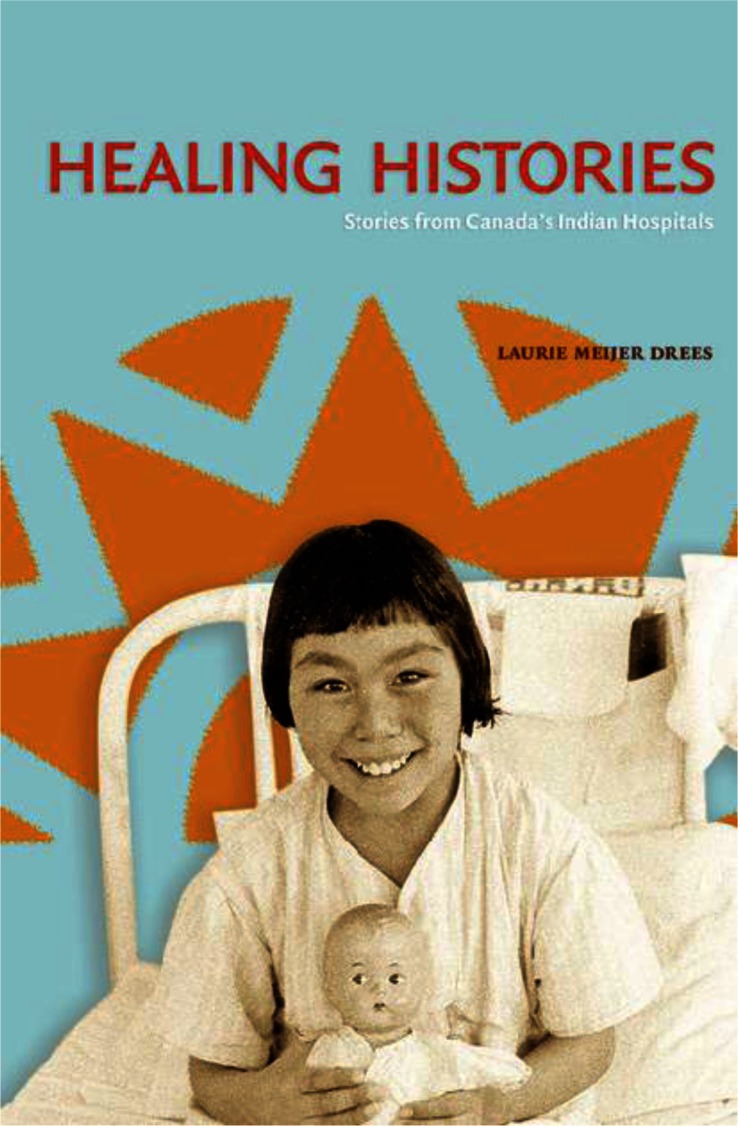


*Healing Histories* is a detailed collection of Aboriginalperspectives on the history of tuberculosis in Canada's indigenous communities and on the federal government's IndianHealth Services. Featuring oral accounts from patients, families,and workers who experienced Canada's Indian Hospital System, it presents a fresh perspective on health care history that includesthe diverse voices and insights of the many people affected bytuberculosis and its treatment in the mid-twentieth century. *Healing Histories* is essential reading for those interested in Canadian Aboriginalhistory, history of medicine and nursing, and oral history.

The book can be ordered from:

http://www.uap.ualberta.ca/UAP.asp?LID=41&bookID=1035

